# Psychometric study of the political violence support scale in a sample of Chilean university students

**DOI:** 10.1186/s40359-026-04416-6

**Published:** 2026-04-01

**Authors:** Gustavo Troncoso-Tejada, José Gálvez-Nieto, Ignacio Norambuena-Paredes, David González Casas

**Affiliations:** 1 Programa de Doctorado en Ciencias Sociales, Universidad de La Frontera, Temuco, Chile; 2 Departamento de Trabajo Social, Universidad de La Frontera, Temuco, Chile; 3 Departamento de Trabajo Social y Servicios Sociales, Universidad Complutense de Madrid, Madrid, Spain

**Keywords:** Political violence, University students, Chile, Psychometric study, Authoritarianism

## Abstract

This study aimed to evaluate the psychometric properties of reliability and validity of the Political Violence Support (PVS) scale in a sample of Chilean university students. A cross-sectional design and non-probabilistic convenience sampling were employed, involving 480 participants (61% women, 37.8% men, and 1.2% other gender), aged between 18 and 45 years (M = 21.35; SD = 2.93). The results obtained through structural equation modelling confirmed the unidimensionality of the PVS, with adequate fit indicators and satisfactory levels of reliability, as indicated by internal consistency. Criterion validity showed positive and significant correlations of small effect size with all dimensions of the Left-Wing Authoritarianism (LWA) scale and with one dimension of the Right-Wing Authoritarianism (RWA) scale. Furthermore, factor invariance analyses provided evidence of configural, metric, and scalar equivalence across sex and age groups. Collectively, the results support the use of the adapted version of the PVS for assessing attitudes that support political violence among Chilean university students.

**Supplementary Information:**

The online version contains supplementary material available at 10.1186/s40359-026-04416-6.

## Introduction

Political violence constitutes a complex and alarming phenomenon in the contemporary world, with profound implications for both the stability of democratic systems and the psychosocial well-being of individuals [1]. Globally, acts of violence motivated by political, social, or religious causes continue to pose a significant threat, resulting in human losses, social polarisation, and distrust towards institutions [2–4]. In this context, various reports warn that in democracies, political violence not only persists but has increased. According to data from V-DEM, the frequency and severity of non-state political violence increased by 26% in democratic countries in recent years [5].

Faced with this panorama, it is necessary to expand the theoretical frameworks and empirical tools that allow for an understanding of the factors that predispose people to accept, justify, or even participate in violent actions. Along these lines, Piazza [5] warns that the rhetoric used by political leaders, as well as the circulation of news and political information, can intensify polarisation and, thereby, exacerbate political violence. Therefore, it is a priority to explore strategies aimed at mitigating polarisation in order to reduce the risks it represents for security and social stability.

In Latin America, and particularly in Chile, recent history has been marked by episodes of social conflict, mass protests, and a growing climate of political polarisation [6]. These processes show that, alongside legitimate forms of democratic mobilisation, attitudes of tolerance toward violence also emerge as a mechanism to promote political or social demands. Understanding the psychosocial conditions that facilitate these attitudes is especially relevant in contexts where perceptions of inequity, alienation, and social exclusion play a central role in the daily lives of citizens [1], which in turn highlights the need for adequate tools to measure them.

The university group constitutes a key setting for analysis. Young adults often occupy a central place in social and political mobilisation due to their vital stage of identity construction, their sensitivity to injustice, and their greater disposition to activism [4, 7]. In Chile, expressions of youth political mobilisation have acquired a sustained prominence in recent decades [8, 9].

Milestones such as the "mochilazo" (2001), the "Penguin Revolution" (2006), the university student movement (2011), and, more recently, the feminist and anti-social inequality protests (2017–2019), illustrate how the student sphere has consolidated as a privileged space for political debate and collective action [10, 11]. These forms of participation are also characterised by their horizontality, autonomy from traditional political parties, and an intensive use of social media as an organisational and visibility tool [12, 13].

In this way, universities have not only been breeding grounds for the organisation and articulation of social demands, but also territories where certain sectors have found narratives that justify the use of violence as a strategy for sociopolitical transformation [14–16], which reinforces the importance of having instruments that allow for the evaluation of how these attitudes are configured in the youth population.

In this context, having valid and reliable psychometric instruments to evaluate support for political violence is crucial. In this regard, the 3 N model of radicalization, which integrates the dimensions of Need, Narrative, and Network, offers a solid theoretical framework for understanding this phenomenon, while also proposing the Political Violence Scale (PVS) as a key tool for measuring the willingness of individuals to accept or justify violence for ideological purposes [1, 17].

Research on political violence support is of strategic importance due to its implications for social cohesion [18–20] and democratic governance [21–23]. The justification of the use of violence as a mechanism for political change could erode trust in institutions, accentuate polarisation, and perpetuate cycles of conflict [5, 24–26].

Likewise, contributions from social psychology indicate that processes such as dehumanisation or moral disengagement influence the decrease of empathy and democratic values [27] and facilitate the legitimisation of violent practices [1, 28]. The foregoing is important, given that empathy and democratic values play a central role in counteracting the individual and social risks that can lead to radicalisation trajectories in youth [29, 30].

A study by Feddes et al. [31] suggests that interventions designed to strengthen empathy in young people can reduce the approval of political violence, highlighting empathy's role as a protective factor against the trajectory toward radicalisation. However, this study has methodological limitations and requires cautious interpretation [27], which makes broadening the observational scope and methodological tools for analysing the phenomenon of support for political violence increasingly important.

Studying this phenomenon in Chilean university students is especially relevant, as this population has a historical tradition of leadership in social mobilisation [32, 33], and they have been exposed to intense political narratives while simultaneously being vulnerable to processes of radicalisation [7].

Therefore, advancing in the study of this phenomenon contributes to a discussion with international literature, through the comparison of radicalization processes present in Chile [34–36] to others reported in Latin America [37, 38], Europe [39–42], and North America [39, 40, 43], thereby enriching the explanatory scope of the 3 N model [1]. This perspective not only expands the comparative framework but also provides valuable input for the design and strengthening of educational policies and ministerial programs aimed at democratic coexistence.

In this context, it is essential to define specifically the concept of political violence and some of its associated theoretical approaches. All violence refers to power relations and disputes over social control, but political violence, in the strict sense, implies the deliberate use of force, threat, or coercion with political, social, or religious motives, aimed at preserving, modifying, or destroying an established social or state order, [44–46]. Likewise, it can be understood as an intentional collective action that resorts to force against people or objects for political reasons, with the purpose of confronting or weakening a group defined by its social identity, such as political parties, nations, social classes, or religious groups [27, 47].

Political violence is understood, within the framework of radicalization processes, as the result of a sequential and progressive development in which individuals or groups are not born radical, but rather go through phases of change in their beliefs, emotions, and behaviors that lead them to justify and eventually execute violent actions for political ends [47–49]. This process, which does not necessarily occur in a linear fashion, involves the gradual transformation of individual, group, and contextual factors, especially the evolution of beliefs and emotions, into forms of legitimization and practice of violence aimed at confronting, modifying, or destroying external groups or the current political system [49–51].

Likewise, political violence is characterised as a collective process that involves mutual coercion between actors, framed within political systems where it affects people and property [52]. From social psychology, it is understood as a learned and rational action, influenced by social ties, structures, and processes, in which existing inter- and intragroup differences determine the roles of both perpetrators and victims [52–54]. The foregoing implies a dispute whose objective is to dominate, modify, maintain, or destroy the previously established social order [45, 55], with its primary defining feature being the intention to cause harm [56, 57].

Therefore, political violence cannot be understood solely as the product of a rational decision, but rather as the result of complex psychosocial processes in which various dimensions converge. At the individual level, it is related to experiences of alienation, loss of meaning, resentment, or perception of injustice. At the contextual level, phenomena such as political polarisation, institutional crises, or geopolitical conflicts come into play. Finally, at the group dimension, social identity, the influence of radicalised networks, and peer legitimisation become relevant, reinforcing violent discourses and practices [1, 17].

This network of individual, contextual, and collective factors that shape political violence, therefore, requires integrative approaches capable of explaining the complexity of its legitimisation, especially among youth exposed to narratives of social change and intensified polarisation. In this sense, the 3 N model [1], a widely used approach, makes it possible to analyse political violence perpetrated by non-state actors seeking to promote political or ideological causes in order to generate social change, as well as to analyse the phenomenon of support for political violence in today’s tense and highly mobilised democratic contexts [4, 58].

The available empirical evidence shows that the Political Violence Support Scale, developed within the framework of the 3 N model [1], is a psychometrically solid instrument validated in Canadian, Pakistani, and Spanish populations. This tool makes it possible to distinguish between individuals who perceive violence as necessary to promote social change and those who prefer to legitimize non-violent strategies [1, 4]. Although this mechanism is replicated across different cultures, relevant contextual variations are recognized between Western and Asian societies, which reinforces the importance of adapting and validating tools in each specific setting to better understand the local particularities of support for political violence.

From this perspective, the application of the Political Violence Support Scale in contexts different from its original validation context, such as the Chilean case, acquires strategic value for the empirical analysis of the phenomenon. In recent years, Chile has presented a scenario characterised by marked political polarisation [59, 60], growing institutional distrust [61–63], and active youth participation in the student sphere [64, 65], transforming the university student body into a key group for examining predisposition toward political violence.

In line with this, it is important to broaden the scope of observation regarding support for political violence in Latin American contexts, specifically the Chilean one, which in recent years has been marked by a climate of post-protest polarisation from 2019–2025 [6]. Although the Political Violence Support Scale has been structurally validated in Canadian, Pakistani, and Spanish contexts [1], a methodological and empirical gap persists in Latin American youth exposed to high social conflictivity. Therefore, there is clear relevance in providing a cross-cultural structural validation of the scale among Chilean university students.

The Political Violence Support Scale constitutes a particularly relevant contribution in this regard, since, unlike other available instruments, it focuses on measuring attitudes of justification toward violence in contexts of social and democratic conflict, centring on subjective support for political violence rather than on the psychosocial consequences of possible armed conflicts [1].

Other previous measurement tools have addressed complementary dimensions of political violence. For example, the Political Life Events Scale for Youth (PLE-Y) evaluates adolescents’ exposure to political violence events and their emotional and behavioural effects [66]. In line with this perspective, the Unction Impairment Scale measures child functional impairment in contexts of international violence [67]. On the other hand, instruments such as the VERA-2 allow for the assessment of the risk of violent radicalisation in adolescents and adults [68], while intention scales such as the Activism-Radicalism Intention Scales (ARIS) examine predisposition to political participation and its degree of radicalisation [69, 70]. In addition, research on the psychosocial trauma caused by political violence has been carried out in diverse contexts such as Northern Ireland, El Salvador, Colombia, and Chile, highlighting the need to develop comparable tools that foster cross-cultural analysis of the phenomenon [71, 72].

The Political Violence Support Scale constitutes a validated and reliable scale, offering a fundamental framework for analysing the justification of violence [1, 46] in contexts of high polarisation and youth mobilisation, such as the Chilean case [73]. This instrument allows for a deeper understanding of the psychosocial dynamics of political violence and opens the door to linking it with relevant psychological constructs, particularly authoritarianism, whose different types, right-wing and left-wing, play key roles in the legitimisation and promotion of violent attitudes.

Authoritarianism is a central psychological construct described in the literature for explaining violent tendencies and intolerant attitudes. From political psychology, it has been conceptualised as a trait that can take on different ideological expressions, the most studied being right-wing authoritarianism [74] and, more recently, left-wing authoritarianism [75]. Both approaches offer key empirical frameworks for its measurement and comparative analysis.

Right-Wing Authoritarianism (RWA) is defined as an attitudinal structure characterised by submission to legitimate authorities, aggression toward those perceived as threats to the social order, and adherence to conventionalism [74, 76, 77]. Various studies have found consistent associations between RWA and the legitimisation of political violence, with significant correlations in countries like Poland and the United States, and mediated associations in Hungary [78–80]. Furthermore, these attitudes tend to intensify in contexts of threat, for example, after terrorist attacks, increasing both support for authoritarianism and political violence [81].

Furthermore, the dehumanisation of out-groups has been identified as a key mediator between Right-Wing Authoritarianism (RWA) and support for punitive and violent measures [82]. RWA also interacts with psychological and social vulnerabilities, such as loneliness and Social Dominance Orientation (SDO), amplifying the predisposition to the use of violence and radicalisation [83]. In the Chilean context, characterised by high political polarisation and institutional distrust [84], RWA could facilitate the legitimisation of state and institutional violence, reinforcing the social acceptance of repressive practices during moments of crisis and instability.

Left-Wing Authoritarianism (LWA) is a construct that has gained relevance for describing a pattern of cognitive rigidity, intolerance, and a disposition toward coercion within progressive ideological frameworks [75, 85]. It is comprised of three main dimensions: Anti-Hierarchical Aggression, Anti-Conventionalism, and Top-Down Censorship, which reflect intolerance toward dominant power structures, internal intolerance toward dissent, and the tendency toward punitive censorship of behaviours contrary to the ideology [75]. Studies conducted in Poland [79] and the United States [86] have shown an association with the acceptance of political violence. Another study has also linked it to traits such as subclinical psychopathy and low levels of empathy [87].

Therefore, studies on political violence support, also integrate authoritarian processes, highlighting both Right-Wing Authoritarianism (RWA) and Left-Wing Authoritarianism (LWA), which, despite their ideological differences, share patterns of cognitive rigidity, ideological obedience, and hostility toward groups perceived as threats. The importance of this measurement is accentuated in youth populations, where factors like social alienation and frustration increase the predisposition to accept moralising narratives that justify political violence, especially in contexts of social mobilisation [15, 88, 89].

Cross-cutting psychological phenomena also influence the legitimisation of political violence. Moral disengagement, which allows harmful acts to be justified without experiencing guilt, is one of the central mechanisms [28]. Added to this is the dehumanisation of social or political adversaries, which reduces empathy and normalises aggression [90]. Likewise, exposure to conspiracy theories intensifies the perception of threat and reinforces paranoid narratives [91]. These processes traverse ideological divisions and are linked to patterns of authoritarian behaviour and the devaluation of democratic values, allowing both conservative and progressive sectors to justify political violence as a means of social transformation or identity defence [74, 86, 92].

In light of this background, it is fundamental to broaden the application of instruments to measure support for political violence in contexts like the Chilean one, characterized by structural inequality and social anomie that can favor its legitimization as a form of resistance or demand for justice [73]. In this sense, the present study aimed to evaluate the psychometric properties of reliability and validity of the Political Violence Scale (PVS) in a sample of Chilean university students. Thus, the goal is to contribute to the methodological strengthening of the field and a better understanding of the factors that predispose to the acceptance or justification of political violence in university youth, also providing relevant evidence for Spanish-speaking contexts in situations of democratic crisis.

## Method

### Participants

The sample consisted of 480 university students, selected through a non-probabilistic sampling method. Of the total, 61% identified as female, 37.8% as male, and 1.2% as another gender. The ages of the participants ranged from 18 to 45 years (M = 21.35; SD = 2.934). In territorial terms, 82.6% of the participants resided in urban areas, while 17.4% lived in rural areas. Regarding their field of study, the majority came from the Social Sciences, Education, and Arts (55.9%), followed by Health (15.2%), and to a lesser extent, Engineering and Technology (8.1%). The remaining groups correspond to Other/Unclassifiable (18.3%), Sciences (1.9%), and Agronomy (0.6%). In relation to civic-social orientation, it is mainly concentrated in those who do not identify with any political position at 30.3%, followed by those who place themselves on the left at 24.7% and the centre-left at 20.3%; to a lesser proportion is the centre at 6.7%, while the right and centre-right positions each reach 0.9%. Finally, regarding ethnic affiliation, 27.6% declared belonging to an ethnic group, while 74.4% indicated they did not identify with any (Table 1). 

**Table 1:** Sociodemographic characterisation of the sample

Variables	Categories	n (%)
Gender	Female	61%
	Male	37.8%
	Other	1.2%
Area of residence	Urban	82.6%
	Rural	17.4%
field of study	Social Sciences, Education and Arts	55.9%
	Engineering and technology	8.1%
	Health	15.2%
	Sciences	1.9%
	Agronomy	0.6%
	Other/Unclassifiable	18.3%
Civic-social orientation	No political position	30.3%
	Left	24.7%
	Center-left	20.3%
	Right	0.9%
	Center-right	0.9%
	Center	6.7%
Ethnic group	No	72.4%
	Yes	27.6%

### Instruments

Four instruments were used in this study: a sociodemographic questionnaire, the Political Violence Support Scale (PVS), the abbreviated version of the Left-Wing Authoritarianism Scale (LWA-9), and the Tripartite Right-Wing Authoritarianism Scale (RWA).

First, a sociodemographic questionnaire with closed-ended questions was administered to gather information on age, sex, gender, family origin (urban/rural), ethnic origin, civic-social orientation, university, program of study, and year of study. This instrument allowed for the collection of basic information about the participants. Its purpose was to characterise the sample and allow for the analysis of possible sociodemographic differences in the study's variables.

The Political Violence Support Scale (PVS) [1] measures the motivational trajectory from political violence to the desire to join radical groups with an ideological basis. The scale consists of 6 items, each answered using a 6-point Likert scale, ranging from 1 (strongly disagree) to 6 (totally agree). The scale is unifactorial and refers to the proclivity and propensity of individuals to accept violence to promote an ideological cause, e.g., ‘*Violence is necessary for social change*’. This instrument presented psychometric quality indicators reported in the original study [1] with an *n* = 235, presenting evidence of validity that supports its psychometric factorial structure (χ2 (gl = 1) = 0.09, p = 0.76, CFI = 1.00, TLI = 1.00, RMSEA = 0.00, SRMR = 0.002.). Additionally, satisfactory reliability indicators are observed (a = 0.83).

The Left-Wing Authoritarianism (LWA) Scale [75] is a 39-item self-report scale answered using a 7-point ordinal response scale (1 = strongly disagree, 7 = strongly agree). The abbreviated version of the Left-Wing Authoritarianism Scale (LWA-9) [85], derived from the original scale by Costello and Patrick [75], was used. The scale presents a factorial structure of three correlated factors named: Anti-Hierarchical Aggression (3 items, e.g., ‘1. *The rich should be stripped of their belongings and status.*’), Top-Down Censorship (3 items, e.g., ‘4. Deep down, just about all conservatives are racist, sexist, and homophobic.’), and Anti-Conventionalism (3 items, e.g., ‘7. *I am in favor of allowing the government to shut down right-wing internet sites and blogs that promote nutty, hateful positions.*’). This scale (LWA-9) showed evidence of validity that supports the three-correlated-factor structure in the sample of Chilean students (*n* = 415), achieving an excellent factorial fit in the CFA and ESEM models (CFI = 0.99; TLI = 0.99; RMSEA = 0.05; SRMR = 0.02). Furthermore, the LWA-9 scale showed adequate levels of internal consistency in its three dimensions: Anti-Hierarchical Aggression (α = 0.85; ω = 0.86; GLB = 0.88), Anti-Conventionalism (α = 0.91; ω = 0.91; GLB = 0.94), and Top-Down Censorship (α = 0.82; ω = 0.82; GLB = 0.87).

The Tripartite Right-Wing Authoritarianism (RWA) Scale measures right-wing authoritarianism (RWA) and was adapted to the Chilean context [93] based on the original version by Duckitt et al. [94] and validated in various international contexts (New Zealand, United States, Israel, and Romania). The Tripartite Right-Wing Authoritarianism (RWA) Scale arises in response to several criticisms directed at the unidimensional approach of the original construct [74]. In later studies, a two-dimensional structure of the RWA has been reported in Argentina [95], and multidimensionality in Colombia [96]. Furthermore, the problem of ambiguity in the length of some items, which made their classification difficult, has been addressed [94, 97]. The RWA [93] consists of 12 items, as assessed in a sample of the Chilean population with a sample size of *n* = 341. The scale is answered using a 6-point ordinal response format (1 = completely disagree, 6 = completely agree), with higher scores indicating higher levels of right-wing authoritarianism. The scale is composed of the following three factorial structures to measure right-wing authoritarianism: 1) Conservatism, which includes 4 items that reflect respect for and obedience to the established order and authorities, e.g., ‘*Obedience and respect for authority are the most important virtues children should learn*’; 2) Traditionalism, which includes 4 items that measure adherence to traditional values and customs, e.g., ‘*The radical and sinful new ways of living and behaving of many young people may one day destroy our society*’; and 3) Authoritarianism, which includes 4 items that evaluate support for coercive and punitive measures to maintain social order, e.g., ‘*Being kind to loafers or criminals will only encourage them to take advantage of your weakness, so it′s best to use a firm, tough hand when dealing with them*’. The RWA [93] presents good psychometric indicators,the confirmatory factor analysis reported a structure of three correlated factors with good fit indices (CFI = 0.98, TLI = 0.98, SRMR = 0.04, RMSEA = 0.04), which supports the construct's validity and its utility for measuring complex sociopolitical phenomena in the current Chilean context. Furthermore, it reported acceptable reliability indices for the three factors: Conservatism (α = 0.86), Traditionalism (α = 0.79), and Authoritarianism (α = 0.80).

### Procedures

The study was approved by the Scientific Ethics Committee of the Universidad de La Frontera (UFRO registration no. 021_25). To administer the instrument, the authorities of the participating universities were contacted, who granted institutional consent. Following the methodological criteria of Muñiz et al. [98], a cross-cultural adaptation was conducted, which included translation and back-translation by independent translators, a panel of expert judges who performed a linguistic review of the scale's items to adapt colloquial expressions and ensure comprehension by the target population [99], and finally a pilot test (*n* = 30 students) [100].

Data were collected using a digital questionnaire on the QuestionPro platform. The research team distributed invitations to the participants via email, which included a link to the questionnaire and the informed consent form. This document explained the objectives of the study, the voluntary nature of participation, the confidentiality and anonymity of the information, and the right to withdraw at any time.

Participants responded to four instruments: a sociodemographic questionnaire, the Political Violence Support Scale, the Left-Wing Authoritarianism Scale, and the Right-Wing Authoritarianism Scale. After data collection, the database was cleaned by removing incomplete records, duplicate responses, and those with implausible response patterns. Only cases with complete data on the main variables were included in the analysis.

### Data analysis

Data analysis was carried out in several successive stages. First, descriptive statistics (mean, standard deviation, skewness, and kurtosis) were calculated for the original items of the Political Violence Support (PVS) scale, using SPSS v.25 software. Subsequently, a confirmatory factor analysis (CFA) model was estimated, given that the PVS has a previously established factorial structure in the literature [1] and the study's objective was to evaluate the replicability of said model in the analysed sample, using the MPLUS version 8.1 program [101], applying the Unweighted Least Squares Mean and Variance adjusted (ULSMV) estimator, which is appropriate for ordinal variables and the absence of normality [102]. The model's goodness of fit was evaluated using the Chi-square (χ^2^), Comparative Fit Index (CFI), Tucker-Lewis Index (TLI), and Root Mean Square Error of Approximation (RMSEA) indices, considering CFI and TLI values above 0.90 and an RMSEA value below 0.08 as indicators of an optimal fit, as proposed by Browne and Cudeck [103]. To evaluate the stability of the measure, factorial invariance analyses were performed, which included the following models [104]: M0 configural (equal number of factors), M1 metric (equality of factor loadings), and M2 scalar (equality of thresholds). Factorial invariance was evaluated according to Chen's [105] recommendations, considering the following change criteria: a ΔCFI ≤ 0.01, a ΔRMSEA ≤ 0.015, and a ΔTLI ≤ 0.01 as evidence of invariance between the compared groups. Finally, to estimate the internal consistency of the evaluated dimensions, McDonald's omega (ω), Greatest Lower Bound (GLB), and Cronbach's alpha (α) coefficients were calculated, using JASP version 0.12.2 software [106].

## Results

### Descriptive statistics

The descriptive analysis of the PVS is presented in Table 2. As can be observed, the item with the highest mean was item 5, “We should never use violence as a way to try to change society” (M = 2.95, SD = 1.49); in contrast, item 6, “There are effective ways to change society in Chile besides resorting to violence,” had the lowest mean (M = 2.18, SD = 1.22). Furthermore, the Kolmogorov–Smirnov (K-S) test was applied to evaluate the normality of the item distribution, with results indicating that the distribution of the items deviates significantly from normality.

**Table 2:** Descriptive statistics

Items	M	Sd	g1	g2	K-S Test
It1	2.55	1.15	0.79	0.60	0.206**
It2	2.87	1.39	0.37	−0.71	0.197**
It3	2.68	1.33	0.56	−0.58	0.226**
It4	2.92	1.53	0.30	−1.01	0.188**
It5	2.95	1.49	0.24	−0.98	0.169**
It6	2.18	1.22	1.03	0.58	0.239**

*M* Mean, *Sd* Standard deviation, *g1* skewness, *g2* kurtosis, *K-S* Kolmogorov–Smirnov normality test

^**^p < 0.001

### Factorial structure

To evaluate the factorial structure of the PVS, a CFA was performed with the six items of the scale (Table 3). The goodness-of-fit indices provided satisfactory results (ULSMV-χ^2^ [df = 9] = 20.558; *p* < 0.001; RMSEA = 0.052; 90% CI = 0.022—0.072; CFI = 0.990; TLI = 0.983), supporting the unidimensional structure of the Political Violence Support Scale. The multiple squared correlations (R^2^) ranged from 0.121 (y3) to 0.910 (y5), indicating that the latent factor accounted for between 12 and 91% of the variance in the observed indicators. Likewise, the average variance extracted (AVE) for the factor was 0.456, a value close to the recommended threshold for convergent validity and consistent with the observed standardised factor loadings in the model.

**Table 3:** Standardized factor loadings, standard errors, and squared multiple correlations for the CFA model

Item	λ	SE	R^2^
y1	0.383*	0.034	0.147
y2	0.754*	0.020	0.568
y3	0.348*	0.037	0.121
y4	0.808*	0.018	0.653
y5	0.954*	0.013	0.910
y6	0.580*	0.032	0.336

λ Standardized factor loading, *SE* Standard error, *R*^*2*^ Squared multiple correlation

^*^p < 0.001

### Factorial invariance

Subsequently, two factorial invariance analyses were performed (Table 4). The results showed that the configural model presented an adequate fit for sex, indicating that the factorial structure is equivalent for men and women. When equality constraints were imposed on the factor loadings (metric invariance) and on the item intercepts (scalar invariance), the observed changes in the fit indices were minimal and remained within the recommended criteria, supporting the metric and scalar equivalence of the measurements between these groups.

**Table 4:** Evaluation of measurement invariance across groups

Group	Model	ULS-χ^2^ (df)	RMSEA	CFI	TLI	SRMR	ΔRMSEA	ΔCFI	Decision
Sex	Configural	41.569* (18)	0.074	0.956	0.927	0.043			Accepted
	Metric invariance	47.712* (23)	0.067	0.954	0.940	0.054	−0.007	−0.002	Accepted
	Scalar invariance	52.547* (28)	0.061	0.954	0.951	0.055	−0.006	0.000	Accepted
Age	Configural	41.876* (18)	0.074	0.955	0.925	0.041			Accepted
	Metric invariance	41.152* (23)	0.059	0.964	0.953	0.044	−0.015	0.009	Accepted
	Scalar invariance	54.717* (28)	0.063	0.949	0.946	0.054	0.004	−0.015	Rejected
	Partial Scalar invariance	43.735 (27)	0.051	0.968	0.965	0.046	−0.008	0.004	Accepted

*MLR* Maximum likelihood estimation with robust standard errors, *χ*^*2*^ Chi-squared, *df* degrees of freedom, *RMSEA* Root mean square error of approximation, *CFI* Comparative fit index, *TLI* Tucker-Lewis index, *SRMR* Standardized root mean square residual, *ΔRMSEA* Change in RMSEA between nested models, *ΔCFI* Change in CFI between nested models

^*^ *p* < 0.01. Measurement invariance by sex was evaluated for male and female participants. The “other gender” category (*n* = 6) was not analyzed separately due to the small sample size

In the case of age, both the configural and metric models also showed adequate fit indices, indicating a comparable factorial structure across age groups. However, the scalar model exhibited a change in the CFI index (ΔCFI = − 0.015) relative to the metric model, slightly exceeding the recommended criterion. Therefore, a partial scalar invariance model was estimated, freeing the threshold of item 3 between age groups. This model showed an adequate fit (CFI = 0.968; RMSEA = 0.051; TLI = 0.965), supporting the presence of partial scalar invariance across age groups.

### Convergent validity

Pearson bivariate correlations were performed among the PVS, LWA, and RWA scales. As presented in Table 5, the results show statistically significant, positive correlations of small effect size (r = 0.149 to 0.242) between the PVS and the LWA factors. Regarding the RWA, it was observed that only the Traditionalism (TRAD) factor correlated negatively, weakly, and statistically significantly with the PVS (r = −0.093, *p* < 0.05). In contrast, the Conservatism (CON) and Authoritarianism (AUTH) factors did not show statistically significant associations with the PVS (*ns*).

**Table 5:** Correlation matrix between PVS, LWA and RWA

Factors	AHA	AC	TDC	PVS	CON	TRAD	AUTH
AHA	1						
AC	0.572**	1					
TDC	0.365**	0.577**	1				
PVS	0.242**	0.189**	0.149**	1			
CON	−0.116*	−0.295**	−0.152**	−0.017 ns	1		
TRAD	−0.214**	−0.406**	−0.306**	−0.093*	0.583**	1	
AUTH	−0.253**	−0.318**	−0.219**	0.038 ns	0.408**	0.430**	1

*PVS * Political violence scale, *LWA* Left-Wing Authoritarianism, factors, *AHA* Anti-Hierarchical Aggression, *AC* Anti-Conventionalism, *TDC* Top-Down Censorship, *RWA* Right-Wing Authoritarianism, factors: *CON* Conservatism, *TRAD* Traditionalism, *AUTH* Authoritarianism

^**^p < 0.01, *p < 0.05, ns = not significant

### Reliability

Once the unidimensional structure of the Political Violence Scale (PVS) was confirmed, the instrument's reliability was analysed. McDonald's ω coefficient was 0.77 (95% CI [0.74—0.80]), while Cronbach's alpha reached a value of 0.74 (95% CI [0.70—0.77]). Likewise, the Greatest Lower Bound (GLB) obtained a higher value of 0.83 (95% CI [0.80—0.86]). Taken together, these results indicate that the PVS has acceptable reliability for its use in research.

## Discussion

The present study aimed to evaluate the psychometric properties of validity and reliability of the Political Violence Support (PVS) scale in a sample of Chilean university students. The findings provide consistent but small-to-moderate evidence regarding the scale's validity and reliability, establishing it as a relevant instrument for examining attitudes of acceptance or justification of political violence in youth and university contexts [1, 107]. These results expand the available knowledge in the Latin American literature and reinforce the importance of having culturally adapted measures to understand phenomena of radicalisation and political polarisation in Chile.

The relevance of the instrument lies in the fact that the PVS underwent an exhaustive process of linguistic evaluation and cultural adaptation, ensuring that its items adequately reflected the language and particularities of the Chilean context. This process of semantic equivalence is crucial to ensure the relevance of the measurement and its cross-cultural validity, allowing for comparisons with international research using the scale in other countries [108, 109]. In this sense, the PVS is positioned as a robust psychometric resource for analysing support for political violence in subjects who have experienced processes of social mobilisation within the democratic system [6, 46, 110].

The results of the confirmatory factor analysis consistently support the unidimensional structure proposed in the original version of the PVS. The fit indices are within the values considered optimal in the psychometric literature, which supports the structural validity of the instrument [1]. This finding is relevant as it confirms that the construct of support for political violence can be represented as a coherent and stable latent dimension in the Chilean case, which lends soundness to its use in future research [111, 112].

Regarding convergent validity, the PVS showed statistically significant correlations of small effect size [113] with the dimensions of left-wing authoritarianism (LWA). This pattern confirms what has been reported in other contexts, where the justification of political violence is linked to progressive authoritarian dispositions [114–116]. Likewise, despite this theoretically expected but limited-magnitude relationship, these results are consistent with expectations in psychological and social research, where constructs are typically complex and influenced by multiple factors [117],nonetheless, they invite future studies with larger samples to replicate these findings. In contrast, the relationship with right-wing authoritarianism (RWA showed limited and ambiguous evidence of criterion validity, restricted to a marginal but significant negative correlation only with Traditionalism (r = −0.093, *p* < 0.05), consistent with the lower ideological affinity between progressive political violence and traditional conservatism [76].

These results pave the way for expanding the study of political violence in the Chilean context, especially in its relationship with left-wing authoritarian stances, thus opening a relevant field for comparative research [118, 119]. Regarding reliability, the PVS reached satisfactory indices in all the estimations used. These results confirm that the scale possesses adequate internal consistency and stability in its measurements, meeting international psychometric standards [1]. The convergence of different reliability coefficients reinforces the robustness of the instrument and its applicability in social and political research in university and youth populations.

This study provides empirical evidence that contributes to a more precise evaluation of the disposition to support political violence in contexts of higher education, social mobilisation, and high conflict [64, 120, 121]. By combining a rigorous methodological design with confirmatory analysis procedures, the work strengthens the framework of available instruments in Latin America for investigating radicalisation processes [112, 122, 123]. Likewise, the findings enable advancements in understanding the psychosocial factors associated with support for violence, at a historical moment of high political polarisation in Chile [124–126].

The invariance analysis revealed that the PVS maintains configural, metric, and scalar equivalence regarding participants' sex. This finding confirms that the scale measures the same construct consistently in men and women, allowing for legitimate comparisons between these groups [70, 127]. This property is fundamental to ensure the validity of differential analyses in future research, particularly in university populations with increasing gender diversity and for the development of educational policies along these lines.

Regarding age, the results showed partial scalar invariance with ΔCFI = − 0.015, slightly above the usual criterion [128]. This suggests that minor differences may exist in item thresholds between age groups, so age-based comparisons should be interpreted cautiously and replicated in larger, more balanced samples [70]. Although the study focused on young university students, this evidence of partial factorial stability indicates that the PVS may be applicable to older cohorts,however, additional studies are needed to confirm its functioning in broader population samples before drawing firm implications for public policy and prevention programmes.

Future research should be oriented towards the development of longitudinal and comparative studies that allow for the evaluation of the stability of radicalisation trajectories over time, as well as their interaction with emerging sociopolitical transformations, and to analyse how ideological, identity-related, or contextual variables can influence or interact with dispositions towards political violence. Particularly relevant is the analysis of the impact of contemporary phenomena, such as digital polarisation, the circulation of disinformation, and new forms of youth mobilisation, on the processes of legitimising political violence in Latin America.

An additional contribution of this study lies in the possibility of conducting cross-cultural comparisons. The validation of the PVS in Chile contributes to the adaptation efforts in other countries, allowing for a comparison of the trajectories of radicalisation and political violence in different sociopolitical contexts. This comparative dimension is especially relevant in Latin America, where social conflict and institutional fragility pose favourable scenarios for the emergence of ideologically legitimised violent attitudes.

Nevertheless, this study has certain limitations that should be taken into consideration. First, the non-probabilistic sampling restricts the generalisation of the findings to the entire Chilean university population; as Hernández-Sampieri et al. [129] point out, this type of sampling reduces representativeness and limits the extrapolation of the results. In our case, the sample is characterised by an overrepresentation of students from Social Sciences, Education, and Arts, as well as a predominantly left-wing and centre-left ideological distribution, suggesting that the observed patterns may reflect attitudes of specific subgroups rather than the entire Chilean student body, potentially influencing the factor structure and observed correlations with RWA. Therefore, we consider that the results should be interpreted as an approximation of the scale's functioning in this specific segment of the student population and not as a representative estimate of the entire Chilean university population, and they should be interpreted cautiously in ideologically homogeneous contexts. In that sense, the generalisation of these findings is limited to the Chilean university student body with similar sociodemographic and political-ideological profiles and in a particular political juncture. Future studies should employ probabilistic sampling designs and recruit participants from greater disciplinary, territorial, and ideological diversity in order to strengthen the scale's external validity. Additionally, test–retest procedures were not used to examine temporal stability, representing a limitation for assessing the instrument's longitudinal precision. Future research should evaluate the scale's stability over time.

Therefore, it is recommended that future research replicate the study with more heterogeneous samples, greater disciplinary diversity, and probabilistic sampling strategies. Likewise, the cross-sectional nature of the design prevents the evaluation of the temporal stability of attitudes towards political violence. Future research should incorporate longitudinal designs that allow for the observation of the evolution of these dispositions in contexts of political crisis or social transformations.

Finally, it would be relevant to move towards the integration of mixed methodologies that combine the quantitative precision of the PVS with qualitative approaches capable of capturing the complexity of student and youth narratives surrounding political violence, according to the context. This would allow for the identification of discursive and subjective nuances that escape the scope of psychometric instruments, enriching the understanding of the phenomenon and broadening the possibilities for intervention in educational and community settings.

## Conclusions

The present study provides evidence on the validity and reliability of the Political Violence Support (PVS) Scale applied to Chilean university students. However, given the use of a convenience sample with specific ideological and disciplinary profiles, the scale's utility should be understood within the framework of university populations with similar characteristics, which enables extension to new studies with probabilistic, heterogeneous samples, and other segments of the student body and adult population. The results confirm the instrument's unidimensional structure and its internal consistency, reaffirming its utility for measuring attitudes of justification for political violence in a context characterised by high polarisation and youth social mobilisation. Furthermore, the modest relationship between support for political violence and left-wing authoritarianism highlights specific ideological dimensions that accompany these attitudes, broadening the understanding of radicalisation processes in Chile.

This study contributes to the consolidation of the PVS as a robust and culturally adapted psychometric tool, which facilitates cross-cultural comparisons and analysis in democratic contexts under tension. It is recommended to continue with longitudinal research and mixed methodologies to delve deeper into the evolution and complexity of dispositions towards political violence, as well as to expand samples to strengthen the representativeness and applicability of the instrument in different populations.

## Supplementary Information

Supplementary Material 1

## Data Availability

The data supporting the findings of this study are not available because they contain sensitive and private information about the participants. In accordance with principles of confidentiality and ethical protection, their disclosure is not permitted.
