# Supplementary material for: Psychometric study of the political violence support scale in a sample of Chilean university students
Source: BMC Psychol. 2026 Apr 1;14:566. doi: 10.1186/s40359-026-04416-6 (PMC13094188; doi:10.1186/s40359-026-04416-6)
Supplement: Supplementary file 1 — Supplementary Material 1 [file 40359_2026_4416_MOESM1_ESM.docx]

**Supplementary material / Material suplementario**

1. **Political Violence Support Scale** (PVS) used in the present study. / Escala de Apoyo a la Violencia Política (PVS) utilizada en el estudio actual.

| **Political Violence Support Scale (PVS) (Bélanger et al., 2019)** | | | | | | | |
| --- | --- | --- | --- | --- | --- | --- | --- |
| Please indicate the extent to which you agree or disagree with each of the following statements by selecting the option that best represents your opinion. | | | | | | | |
|  |  | Strongly disagree | Disagree | Something at odds | Something agreed upon | Agreed | I completely agree |
| 1 | When using violence to further a just cause, everybody is fair game. | ☐ | ☐ | ☐ | ☐ | ☐ | ☐ |
| 2 | Violence is necessary for social change. | ☐ | ☐ | ☐ | ☐ | ☐ | ☐ |
| 3 | It is acceptable to retaliate against someone who insults my values and beliefs. | ☐ | ☐ | ☐ | ☐ | ☐ | ☐ |
| 4 | I would never consider physical violence to further a just cause (R).* | ☐ | ☐ | ☐ | ☐ | ☐ | ☐ |
| 5 | We should never use violence as a way to try to change society (R).* | ☐ | ☐ | ☐ | ☐ | ☐ | ☐ |
| 6 | There are effective ways of changing society in Canada other than resorting to violence (R).* | ☐ | ☐ | ☐ | ☐ | ☐ | ☐ |
| Note:  * These items are negative; it is suggested that they be recoded for future analysis. | | | | | | | |
|  |  |  |  |  |  |  |  |

| **Escala de Apoyo a la Violencia Política (PVS, por sus siglas en inglés)**  **(Bélanger et al., 2019)** | | | | | | | |
| --- | --- | --- | --- | --- | --- | --- | --- |
| Por favor, indique en qué medida está de acuerdo o en desacuerdo con cada una de las siguientes afirmaciones seleccionando la opción que mejor represente su opinión. | | | | | | | |
|  |  | Muy en desacuerdo | En desacuerdo | Algo en desacuerdo | Algo de acuerdo | De acuerdo | Totalmente de acuerdo |
| 1 | Cuando se usa la violencia para promover una causa justa, cualquiera de nosotros puede salir dañado. | ☐ | ☐ | ☐ | ☐ | ☐ | ☐ |
| 2 | La violencia es necesaria para el cambio social. | ☐ | ☐ | ☐ | ☐ | ☐ | ☐ |
| 3 | Es aceptable tomar represalias contra alguien que insulta mis valores y creencias. | ☐ | ☐ | ☐ | ☐ | ☐ | ☐ |
| 4 | Nunca consideraría la violencia física para promover una causa justa (R).* | ☐ | ☐ | ☐ | ☐ | ☐ | ☐ |
| 5 | Nunca debemos usar la violencia como una forma de tratar de cambiar la sociedad (R).* | ☐ | ☐ | ☐ | ☐ | ☐ | ☐ |
| 6 | Hay formas efectivas de cambiar la sociedad en Chile además de recurrir a la violencia (R).* | ☐ | ☐ | ☐ | ☐ | ☐ | ☐ |
| Nota:  * Estos ítems son negativos se sugiere recodificarlos para futuros análisis. | | | | | | | |
|  |  |  |  |  |  |  |  |

1. Comparative table of items on the Political Violence Support Scale in its original version and the Chilean version / Tabla comparativa de los ítems de la Escala de Apoyo a la Violencia Política en su versión original y la versión chilena.

| **Ítems** | **Original scale in English**  **(Bélanger et al., 2019)** | **Adaptation Chilean version** |
| --- | --- | --- |
| 1 | When using violence to further a just cause, everybody is fair game. | Cuando se usa la violencia para promover una causa justa, cualquiera de nosotros puede salir dañado. |
| 2 | Violence is necessary for social change. | La violencia es necesaria para el cambio social. |
| 3 | It is acceptable to retaliate against someone who insults my values and beliefs. | Es aceptable tomar represalias contra alguien que insulta mis valores y creencias. |
| 4 | I would never consider physical violence to further a just cause (R). | Nunca consideraría la violencia física para promover una causa justa (R). |
| 5 | We should never use violence as a way to try to change society (R). | Nunca debemos usar la violencia como una forma de tratar de cambiar la sociedad (R). |
| 6 | There are effective ways of changing society in Canada other than resorting to violence (R). | Hay formas efectivas de cambiar la sociedad en Chile además de recurrir a la violencia (R). |
